# Editorial: Cognitive interactions and movement adaptations

**DOI:** 10.3389/fpsyg.2026.1856942

**Published:** 2026-05-15

**Authors:** Daniela De Bartolo, Elizabeth Thomas

**Affiliations:** 1 SmArt Lab, IRCCS Santa Lucia Foundation, Rome, Italy; 2 INSERM, CAPS UMR 1093, Universite Bourgogne Europe Polytech Dijon, Dijon, France

**Keywords:** adaptive behavior, aging and mobility, cognitive-motor integration, motor learning and control, perceptual-motor coupling

The present Research Topic brings together 10 contributions that collectively advance our understanding of how cognitive and motor processes interact across development, expertise, fatigue, aging, and technological mediation. Moving beyond the traditional view of cognition as a supervisory system detached from action, the articles in this collection converge on a shared premise: cognition and movement are dynamically co-regulated processes, mutually shaping performance, adaptation, and resilience.

## Physical exercise as cognitive technology

A first group of studies addresses the impact of physical exercise on executive functioning and neurophysiological mechanisms, moving beyond generic claims that “exercise benefits cognition” to specify *how, for whom*, and *through which mechanisms* such benefits emerge.

Wang et al. examined adolescents with obesity and low socioeconomic status and demonstrated that both high-intensity interval exercise (HIIE) and moderate-intensity continuous exercise (MICE) improved inhibition, working memory, and cognitive flexibility, though with distinct performance profiles. MICE appeared particularly beneficial for inhibitory accuracy, whereas HIIE produced stronger effects on flexibility reaction times and BMI reduction.

Si et al. similarly reported executive function improvements following variable-intensity interval training (VIIT) and moderate-intensity continuous training (MICT) in university students. Crucially, behavioral improvements were accompanied by changes in prefrontal hemodynamic (ΔHbO_2_), thereby linking cognitive enhancement to underlying neural activation patterns.

Complementing these findings, Jiang et al. provided meta-analytic evidence that physical training modulates corticospinal excitability in athletes, as measured by transcranial magnetic stimulation (TMS), with sport-specific patterns of neuroplastic adaptation.

Collectively, these research findings have added to the existing literature that views physical exercise as an important factor influencing cognition from a neurobiological point of view (Hillman et al., 2008; Diamond and Ling, 2016). However, importantly, it must be highlighted that these studies have reiterated the need for precision in developing such cognitive-based interventions.

## Motor learning: errors, autonomy, and cognitive load

Another group of related themes revolves around the issue of motor learning, specifically how cognitive load, autonomy, affective factors, and fatigue can impact the process of learning and retaining a particular skill. The experiment by van der Capio et al. suggests the need to look into errorless motor learning among children and elderly persons, separating cognitive aspects like motor variability and prefrontal cortex activity, and affective factors such as perceived competence.

In this line, Akizuki et al. studied self-controlled feedback timing, showing that learner's selection of feedback timings affects intrinsic motivation and workload. This study supports self-determination theory (Deci and Ryan, 2000) by highlighting the importance of autonomy as an essential factor for learning efficiency.

Lastly, Banihosseini et al. expands on this idea by examining errorless (implicit) and errorful (explicit) motor learning when there is mental and physical fatigue. Errorless learning was found to be more resistant to cognitive fatigue induced by the Stroop task and physical fatigue induced by maximal voluntary contraction, consistent with theoretical perspectives predicting less utilization of working memory capacity in implicit tasks (Masters and Maxwell, 2008).

These works collectively indicate that ideal motor learning contexts are those in which cognitive load is controlled, autonomy is cultivated, and resistance is established under challenging circumstances.

## Cognitive–motor interference and ecological validity

Another important area of study within this Research Topic pertains to dual-task paradigms and real-world situations.

In their study of outdoor dual-task walking with a powered exoskeleton for lower limbs, Riedel et al. reveal that the presence of an exoskeleton during locomotion can augment cognitive load demands through mechanisms of task prioritization (preservation of gait variables and deterioration of performance in the cognitive task) and short-term familiarity that lowers perceived workload without negating dual-task costs. The recent work by Cai and Li, resents a valuable counterpart to these studies by offering a unique perspective on cognitive-motor interactions in ecologically valid environments. Through the exploration of the interplay between cognitive processes and movement requirements under naturalistic or more complex stimulus presentations, this research emphasizes the significance of considering not just the interference created by the tasks themselves but also the nature of the stimuli presented.

These results reinforce classic models of attention and gait (e.g., posture-first prioritization; Woollacott and Shumway-Cook, 2002) and systematic evidence that dual-tasking compromises walking under certain conditions (Al-Yahya et al., 2011; De Bartolo et al., 2021), while adding an urgently relevant dimension: assistive technologies must be evaluated not only for biomechanical assistance (De Pasquale et al., 2025), but also for cognitive compatibility in realistic environments (De Bartolo et al., 2026).

## Skill experience, perceptual–motor coupling, and attention

Two contributions broaden the “cognition-in-action” lens by showing how skill experience and structured movement training shape perceptual–motor integration and attentional processing.

Zhang et al. found that the practice of specific Hongquan martial arts influenced visual motor integration and associated factors in children, such as attention and executive function tasks, implying that such practice may be important for cognitive-motor development.

In young adults, Aly et al. show that motor skill experience modulates attentional processing during an auditory oddball task (P3 component), and that these effects are observed regardless of open- vs. closed-skill categorization. By additionally considering aerobic fitness, the study encourages a shift away from simple sport-type dichotomies toward more specific explanatory variables (experience dose, training characteristics, fitness, and task demands).

Together, these findings suggest that motor experience does not remain confined to performance *per se* but extends to broader attentional and perceptual–motor processes. In this sense, movement practice may contribute to shaping the cognitive architecture that supports adaptive behaviour across contexts.

## Aging, navigation, and functional vulnerability

Finally, Kossowska-Kuhn et al. extend cognitive–motor interaction into functional mobility and independence by examining navigation in older adults with and without cognitive impairment. The study reports poorer objective navigation performance and lower subjective memory in cognitively impaired participants relative to a community-dwelling comparison sample, alongside a dissociation in which subjective navigation measures are not reliably predicted by objective navigation performance or subjective memory.

One aspect of the present contribution that aligns with the translational nature of the Topic's research question is a practical application based on this work: when trying to maintain functional independence and mobility in the elderly population, interventions should consider using objective measures rather than self-reporting, along with treating navigation as a cognitive-motor process with real-life consequences. More generally, it must be noted that the results suggest that learning, mobility, and performance cannot be understood in terms of discrete cognitive and motor factors: instead, they involve the continuous adjustment of attention, perception, and executive processes given certain constraints defined by the environment and body. This dynamic interplay of cognitive mechanisms forms the core of many contributions in the present Research Topic, including, for instance, the study of exercise-induced changes in executive control or the analysis of the structure of practice based on error management and autonomy. Indeed, one general idea shared by all the studies considered is that adaptive behaviour is linked to the organization of cognitive resources during action.
